# Development and Validation of an Ultrasound-Based Radiomics Nomogram for Identifying HER2 Status in Patients with Breast Carcinoma

**DOI:** 10.3390/diagnostics12123130

**Published:** 2022-12-12

**Authors:** Yinghong Guo, Jiangfeng Wu, Yunlai Wang, Yun Jin

**Affiliations:** Department of Ultrasound, Dongyang People’s Hospital, No. 60 Wuning West Road, Dongyang 322100, China

**Keywords:** ultrasound, HER2, breast carcinoma, radiomics

## Abstract

(1) Objective: To evaluate the performance of ultrasound-based radiomics in the preoperative prediction of human epidermal growth factor receptor 2-positive (HER2+) and HER2− breast carcinoma. (2) Methods: Ultrasound images from 309 patients (86 HER2+ cases and 223 HER2− cases) were retrospectively analyzed, of which 216 patients belonged to the training set and 93 patients assigned to the time-independent validation set. The region of interest of the tumors was delineated, and the radiomics features were extracted. Radiomics features underwent dimensionality reduction analyses using the intra-class correlation coefficient (ICC), Mann–Whitney U test, and the least absolute shrinkage and selection operator (LASSO) algorithm. The radiomics score (Rad-score) for each patient was calculated through a linear combination of the nonzero coefficient features. The support vector machine (SVM), K nearest neighbors (KNN), logistic regression (LR), decision tree (DT), random forest (RF), naive Bayes (NB) and XGBoost (XGB) machine learning classifiers were trained to establish prediction models based on the Rad-score. A clinical model based on significant clinical features was also established. In addition, the logistic regression method was used to integrate Rad-score and clinical features to generate the nomogram model. The leave-one-out cross validation (LOOCV) method was used to validate the reliability and stability of the model. (3) Results: Among the seven classifier models, the LR achieved the best performance in the validation set, with an area under the receiver operating characteristic curve (AUC) of 0.786, and was obtained as the Rad-score model, while the RF performed the worst. Tumor size showed a statistical difference between the HER2+ and HER2− groups (*p* = 0.028). The nomogram model had a slightly higher AUC than the Rad-score model (AUC, 0.788 vs. 0.786), but no statistical difference (Delong test, *p* = 0.919). The LOOCV method yielded a high median AUC of 0.790 in the validation set. (4) Conclusion: The Rad-score model performs best among the seven classifiers. The nomogram model based on Rad-score and tumor size has slightly better predictive performance than the Rad-score model, and it has the potential to be utilized as a routine modality for preoperatively determining HER2 status in BC patients non-invasively.

## 1. Introduction

Breast carcinoma (BC) is the most common malignancy and the most frequent cause of carcinoma mortality in women worldwide [1], and it is a complex and heterogeneous disease [2,3,4]. Currently, BC is mainly classified into hormone-receptor-positive, human epidermal growth factor receptor 2-positive (HER2+), and triple-negative BC on the basis of histopathological characteristics [5,6].

HER2+ BC, in which the cells do not express estrogen receptors and progesterone receptors, accounts for about 15% of all BC cases and presents a high rate of recurrence and poor prognosis compared with hormone-receptor-positive BC [7,8,9]. Nevertheless, over the last two decades, as agents that target HER2, including trastuzumab and pertuzumab, are extensively applied in clinical practice, significant advances have been made in the treatment of HER2+ BC and overall survival has improved [10,11,12]. Hence, the status of HER2 is one of the most significant and decisive factors in the treatment decision and prognosis for breast carcinoma patients.

So far, the evaluation of HER2 status in breast carcinoma patients mainly relies on immunohistochemistry (IHC) examination after surgical tumor excision or biopsy [13], whereas both biopsy and surgery are invasive procedures and may lead to an increased risk of complications such as seroma, local pain, and infection [14,15]. Moreover, the evaluation results of a few tissue biopsies do not necessarily represent HER2 status of the whole tumor [16]. In addition, in our center, routine histopathological findings are analyzed, but patients still need to spend extra to get results from IHC. Therefore, it is urgent to develop an economical, non-invasive, and precise pretreatment technology to predict HER2 status in breast carcinoma patients.

Radiomics is a new research field on the basis of quantitative imaging methods, which are mainly adopted to extract and analyze a large number of imaging features hardly perceived by radiologists to reflect tissue information [17,18]. Recent studies demonstrate that radiomics features extracted from magnetic resonance imaging (MRI) and computed tomography (CT) images have been widely used in diagnosis, prediction of tumor stage and histological subtype, as well as prognostic evaluation [19,20,21,22]. MRI and CT are limited by economic cost and/or equipment availability. Compared with the above imaging technologies, ultrasound, recognized as a radiation-free, convenient, and reasonably priced technology, is universally used for breast carcinoma screening and diagnosis [23]. A number of researchers have extended radiomics to ultrasound imaging [24,25]. Prior ultrasound radiomics studies have shown that molecular subtypes of BC are related to qualitative imaging characteristics and histopathologic features [26,27].

To the best of our knowledge, there are still relatively few studies to predict HER2 status of breast carcinoma using the method of ultrasound-based radiomics. We hypothesized that ultrasound radiomics features might provide guidance for predicting HER2 status in patients with breast carcinoma and would like to develop and validate an ultrasound radiomics model that could predict HER2 status.

## 2. Materials and Methods

### 2.1. Patient Cohorts

The institutional review board approved this retrospective study, and the requirement for written informed consent was waived.

In total, 522 female patients confirmed as primary BC based on pathology examination by means of biopsy or surgical excision and examined by ultrasound before treatment at our institution from March 2019 to November 2021 were retrospectively collected.

**Figure 1:**
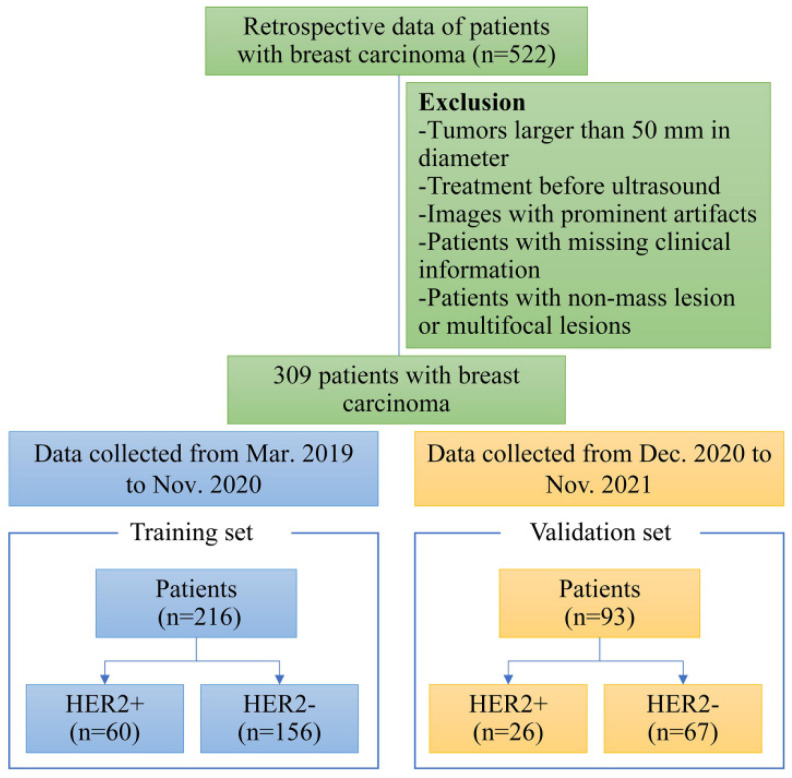
The patient enrollment process for this study.

Exclusion criteria were as follows: (a) ultrasound images not suitable for radiomics study because of poor quality, artifacts, calcifications, or cystic changes (*n* = 48); (b) tumors larger than 50 mm in diameter (incompletely displayed in a single plane) (*n* = 27); (c) patients who underwent biopsy, radiotherapy, and/or chemotherapy before ultrasound examination (*n* = 65); (d) patients with multifocal lesions or non-mass BC (*n* = 4040); and (e) patients with missing clinical characteristics and/or postoperative histopathology (*n* = 32); Finally, there were 309 eligible patients with BC, of whom those from March 2019 to November 2020 served as the training set (*n* = 216), while the remaining patients formed the time-independent validation set (*n* = 93). The flowchart of patient selection is shown in Figure 1.

### 2.2. Pathological Assessment

IHC is the leading clinical technology for immunostaining, which can precisely determine the molecular subtypes of BC with high specificity. The estrogen receptor (ER) and progesterone receptor (PR) status was considered positive if ≥1% of tumor cells had positively stained nuclei [28]. For HER2 status identification, an IHC score 3+ of HER2 was considered as positive, while an IHC score 0 or 1+ of HER2 was considered as negative. An IHC score of 2+ was considered indeterminate, and then fluorescence in situ hybridization (FISH) was carried out to assess gene amplification, and HER2 was classified as positive if the ratio was ≥2.0 [6]. For Ki-67 status, tumors with greater than 14% positive nuclei were considered to have high expression, while other cases were considered to have low expression [29].

### 2.3. Clinical Characteristics

Clinical data such as age, tumor size, and tumor location were obtained from patients’ medical records. Status of ER, PR, and HER2, Ki-67 levels, molecular subtype, lymph node metastasis, and histological type of tumor were obtained by reviewing patients’ pathology reports.

### 2.4. Image Acqusition and Segmentation

Breast ultrasound examinations were carried out by sonographers with more than 5 years of experience in breast ultrasound imaging, within 2 weeks before surgical resection. Ultrasound was performed using the LOGIQ E9 ultrasound system with a 6–15 L linear array probe and the Siemens Acuson S2000 with a 6–18 L linear array probe with radial, transverse, and longitudinal scanning on both breasts. The imaging parameters were consistent among patients: gain was about 50%; image depth was about 3.0 cm to 5.0 cm; and focus paralleled the lesion. The ultrasound image was 1164 × 873 pixels and 1024 × 768 pixels in size on the LOGIQ E9 and Siemens Acuson S2000 devices, respectively. The image of the largest section of the breast tumor with the clearest imaging was saved in the format of Digital Imaging and Communications in Medicine to maximize the preservation of the image information. Manual segmentation was performed on gray-scale ultrasound images of breast lesions. Sonographer 1 (with more than 5 years of experience in breast ultrasound imaging) with no information about the patient’s clinical history selected the largest plane of each breast lesion and drew an outline of the region of interest (ROI) by using ITK-SNAP software (version 3.4.0).

### 2.5. Radiomic Feature Extraction

A total of 788 radiomics features, consisting of shape, statistics, texture, and wavelet features, were extracted. Radiomics features were extracted using the “pyradiomics” package of Python (version 3.7.11). These ultrasound radiomic features were divided into four categories, including 14 two-dimension shape-based features, 18 first-order statistics features, 22 gray-level co-occurrence matrix (GLCM) features, 16 gray-level run length matrix (GLRLM) features, 16 gray-level size zone matrix (GLSZM) features, 14 gray-level dependence matrix (GLDM) features, and 688 features derived from first-order GLCM, GLRLM, GLSZM, and GLDM features using wavelet filter images. Supplementary Material Data S1 contains details on the ultrasound radiomics extraction settings.

### 2.6. Evaluation of Inter- and Intra-Class Correlation Coefficient

The inter- and intra-class correlation coefficients (ICCs) were adopted to test the reproducibility of feature extraction. Sonographers 1 and 2 (both with more than 5 years of experience in breast ultrasound imaging) drew ROIs on the same ultrasound images from the 50 randomly selected patients and extracted the radiomics features. Two weeks later, sonographer 1 repeated ROI segmentation on the same ultrasound images and extracted the radiomics features to assess the intra-observer reproducibility. An ICC greater than 0.75 suggested a good agreement for the feature extraction.

### 2.7. Radiomics Feature Selection

All the radiomics features were standardized by the z-score algorithm to ensure that the scale of feature value was uniform and improve the comparability between features, which was realized in the proportional scaling of the original data. The features with ICCs less than 0.75 were excluded.

In the training set, the Kolmogorov-Smirnov test was first performed to assess whether variances were normally distributed, and Levene’s test was used to assess the equality of variance. An independent sample *t* test was used for variables with a normal distribution and homogeneity of variance. Otherwise, the Mann–Whitney U test was used. The radiomics features that showed no significant differences were excluded. The remaining radiomics features were further screened by using penalized logistic regression with a least absolute shrinkage and selection operator (LASSO) algorithm. An optimal lambda was selected through 10-fold stratified cross-validation, which was tuned to achieve minimum mean square error. Thus, features with a non-zero coefficient in the model were regarded as the most representative features.

### 2.8. Development and Validation of the Prediction Model

The radiomics score (Rad-score) was calculated for each lesion using LASSO regression and a linear combination of the values of the selected features weighted by their respective non-zero coefficients. Based on the Rad-score, seven machine learning classifiers consisting of decision tree (DT), K nearest neighbors (KNN), random forest (RF), support vector machine (SVM), logistic regression (LR), naive Bayes (NB), and XGBoost were used to construct the prediction model in the training set. The classifier with the highest AUC value in the validation set was obtained as the Rad-score model.

### 2.9. Clinical Model and Nomogram Model

Clinical features that showed a statistical difference between the HER2+ and HER2− BC in the training set were adopted to develop the clinical model by using the logistic regression method. In addition, the nomogram model combining significant clinical factors and the Rad-score was constructed for personalized HER2 status prediction.

**Figure 2:**
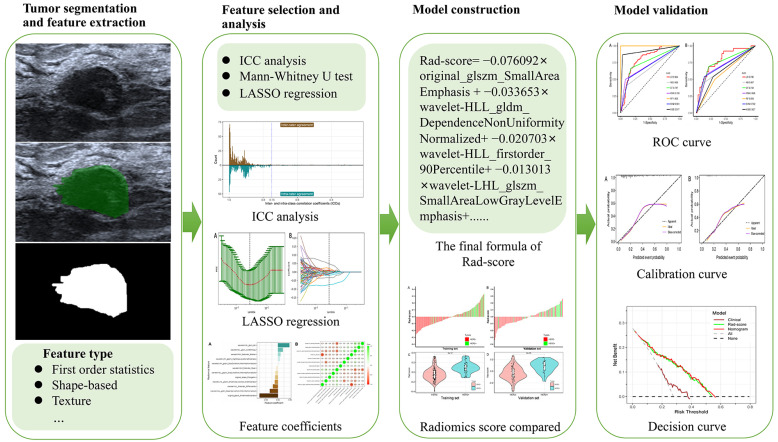
Schematic representation of the radiomics analysis steps.

We evaluated the performances of all the models in the time-independent validation set in terms of sensitivity, specificity, positive predictive value (PPV), negative predictive value (NPV), accuracy, and the area under the receiver operating characteristic (ROC) curve (AUC). To verify the robustness of the nomogram model, the calibration curve [25] was plotted. Furthermore, decision curve analysis (DCA) [26] was also utilized to select the model that maximized patient benefits. The flowchart of this research is shown in Figure 2.

### 2.10. Statistical Analysis

R version 3.5.1 software was used for statistical analysis and figure plotting. Radiomics features were extracted from each ROI using the “pyradiomics” package of Python (version 3.7.11). The continuous variables with normal distribution and homogeneity of variance were shown as the mean (standard deviation) and tested by an independent sample *t* test; otherwise, the data were analyzed by the Mann–Whitney U test and expressed as the median (interquartile range). For categorical variables, the chi-square analysis or Fisher’s exact tests were applied to compare the results. A two-tailed *p* < 0.05 indicated a significant difference.

## 3. Results

### 3.1. Clinical and Pathological Characteristics

The clinical and pathological characteristics of the training and validation sets were compared, and there was no statistically significant difference found (*p* > 0.05) (Table 1). This suggested that the training and validation sets were harmonious in these clinical and pathological characteristics.

### 3.2. Radiomics Feature Extraction and Selection

**Figure 3:**
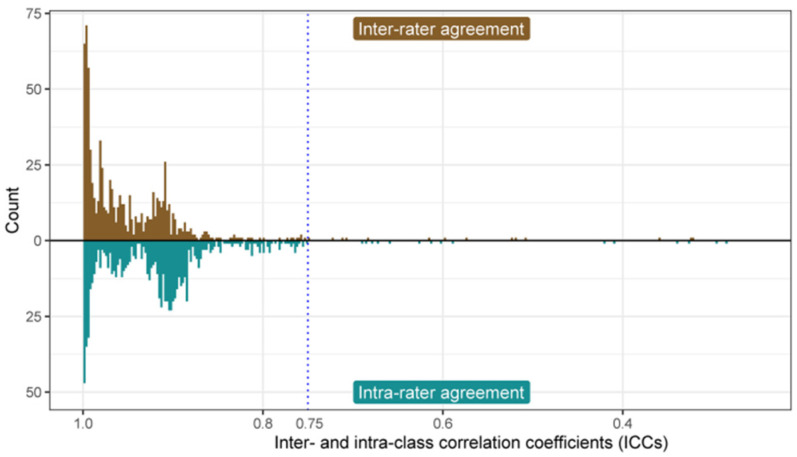
Bar plots of intra- and inter-observer ICC. Upper: inter-rater agreement; Lower: intra-rater agreement. ICC: intra-class correlation coefficient.

A total of 788 radiomics features were extracted from the ultrasound images of each patient. The reproducibility of ultrasound radiomics features extraction was assessed. The intra-observer correlation coefficient of sonographer 1 in two extractions was between 0.296 and 0.996, while the inter-observer correlation coefficient of extraction by sonographer 1 and sonographer 2 was between 0.323 and 0.989. Finally, 23 radiomics features (ICC < 0.75) were excluded. The ICC evaluation results are shown in Figure 3. The morphological characteristics of the randomly selected lesions for ICC assessment are provided as Supplementary Material Data S2. All of the following analyses were based on the radiomics features extracted by sonographer 1.

**Figure 4:**
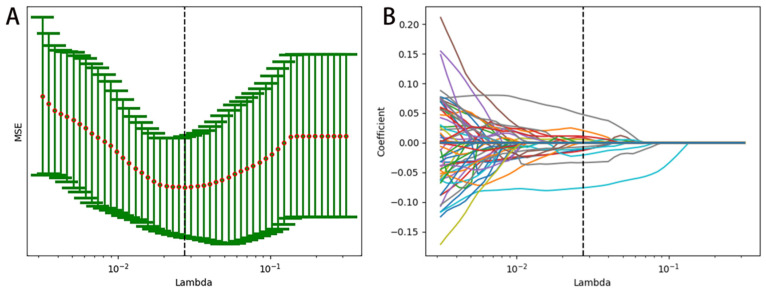
Feature selection and Rad-score building by LASSO. (**A**) A 10-fold cross validation was used to predict mean square error of the Rad-score building by different Lambda values. (**B**) The coefficient profiles of the radiomics features determined by different Lambda values.

In the training set, after evaluating the differences of radiomics features by the Mann–Whitney U test, 321 radiomics features were used for further analysis. Then, the optimum Lambda (Lambda = 0.027464741148160516) was determined for the LASSO regression, and 12 radiomics features with nonzero coefficients were selected to differentiate HER2+ from HER2− BC (Figure 4).

**Figure 5:**
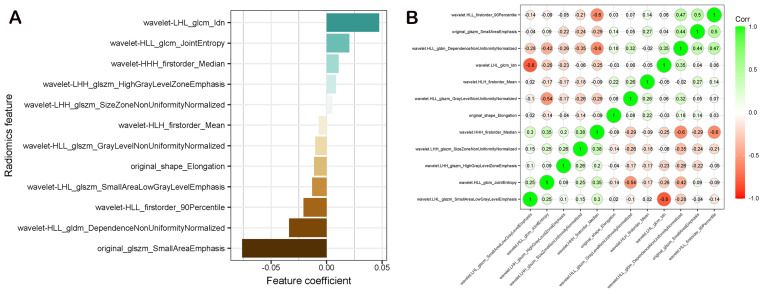
(**A**) The coefficients of radiomics features to construct the Rad-score; (**B**) a Pearson correlation coefficient heatmap of the selected features for predicting HER2 status. Green color denotes a positive correlation, the red color denotes a negative correlation, and the shade of the color indicates the degree of correlation.

Detailed information on the HER2+ BC-related features is shown in Table 2, and the nonzero coefficients of the selected features based on the LASSO regression are shown in Figure 5A. Moreover, the Pearson correlation coefficient between any pair of selected features was computed, and the correlation coefficient matrix heatmap is shown in Figure 5B.

### 3.3. Radiomics Score Calculation

**Figure 6:**
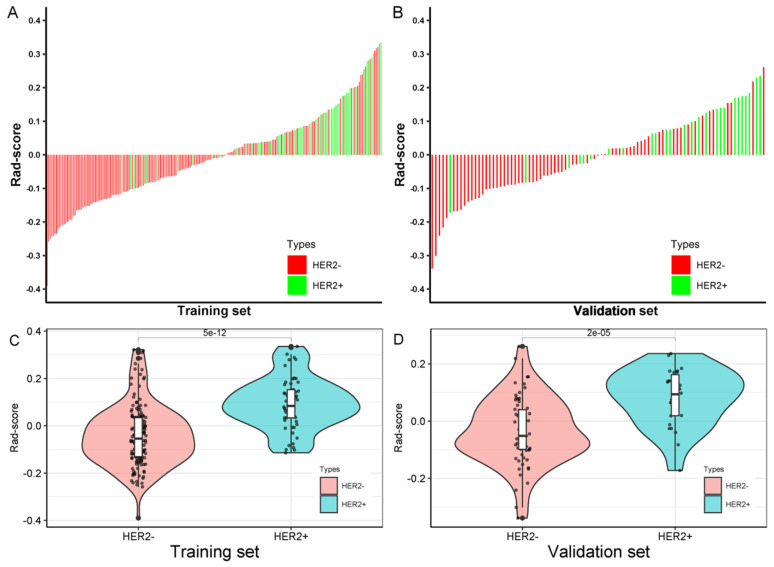
Radiomics score for each breast carcinoma patient in the training (**A**) and validation sets (**B**); Distribution of radiomics score values of the HER2+ and HER2− groups in the training (**C**) and validation sets (**D**).

The radiomics score (Rad-score) for each patient in the training and validation sets was calculated through a linear combination of the nonzero coefficient features based on the LASSO regression, as shown in Figure 6A,B. The corresponding fitting formula is listed in Supplementary Material Data S3. In the training set, the medians of Rad-score showed a statistical difference between the HER2+ and HER2− BC (0.0838 vs. −0.0546, *p* < 0.001), and the same results were achieved in the validation set (0.0936 vs. −0.0518, *p* < 0.001) (Figure 6C,D, Table 3).

### 3.4. Construction and Evaluation of Machine Learning Classifier

Seven machine learning classifiers (KNN, DT, RF, SVM, LR, NB, and XGBoost) were then adopted to develop the prediction model based on the Rad-score. The sensitivity, specificity, accuracy, PPV, NPV, and AUC values of the seven machine learning classifiers are shown in Table 4.

**Figure 7:**
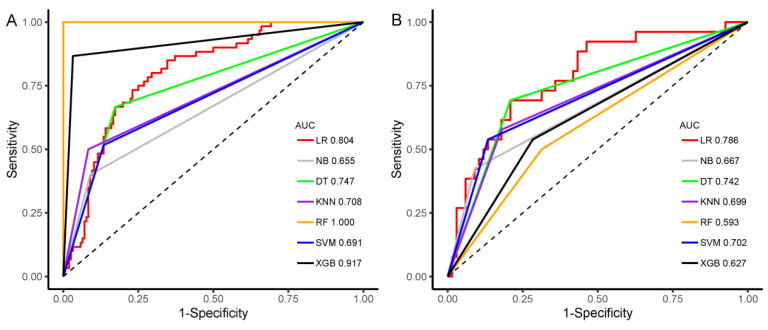
Receiver operating characteristic curves of seven machine learning classifiers predicting HER2+ status in training (**A**) and validation sets (**B**).

Among the classifiers, the general accuracies of the RF and XGBoost were 100.0% and 94.0% in the training set and 63.4% and 66.7% in the validation set, which suggested overfitting. The accuracy was 63.4% in the RF classifier and 77.4% in the SVM and NB classifiers; the AUC values of the seven machine learning classifiers ranged from 0.593 to 0.786 in the validation set, with the LR classifier performing the best and the RF classifier performing the worst. The LR classifier with the highest AUC value was selected as the Rad-score model. In addition, a comparison of the ROC curves of the seven machine learning classifiers in the training set and validation set is shown in Figure 7. Furthermore, the AUC values between any pair of the classifiers were compared, and the *p* values were obtained by DeLong test, which are shown in Table 5.

### 3.5. Clinical Model and Nomogram Model

**Figure 8:**
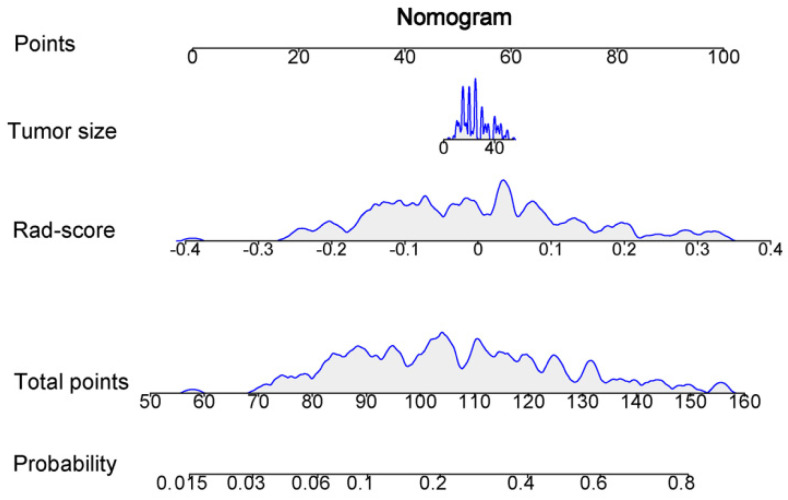
Nomogram based on the combination of the tumor size and Rad-score was developed using logistic regression analysis.

Comparison of the clinical features between the HER2+ and the HER2− BC in the training set was performed. Tumor size (*p* = 0.028) and Rad-score (*p* < 0.001) were the significant factors to distinguish the HER2+ from HER2− BC. Other clinical features such as age, tumor location, ultrasound equipment, and ultrasound-reported lymph node status were not identified as potential factors for predicting the HER2+ type (Table 6). Then, the clinical model based on tumor size was constructed using logistic regression. At the same time, the nomogram model was established by combining the tumor size and Rad-score (Figure 8).

**Figure 9:**
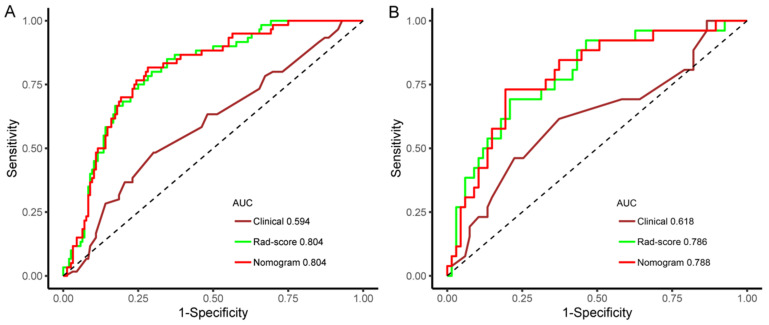
Receiver operating characteristic curves of the three models predicting HER2+ type in the training (**A**) and validation sets (**B**).

Moreover, the predictive abilities of the clinical, Rad-score and nomogram models were compared. The results for each model are summarized in Table 7. The ROC curves of the three models to predict the HER2+ type are shown in Figure 9. In the time-independent validation set, the AUC value of the nomogram was significantly higher than that of the clinical model (AUC, 0.788 vs. 0.618; DeLong test, *p* = 0.016). Although the nomogram model performed slightly better than the Rad-score model, there was no statistically significant difference between them (AUC, 0.788 vs. 0.786; DeLong test, *p* = 0.919).

The LOOCV algorithm was carried out to validate the reliability and stability of the results, which yielded a high median AUC (0.790 in the validation set), indicating that the predictive performance of the nomogram model was reliable and stable.

### 3.6. Model Performance Evaluation

**Figure 10:**
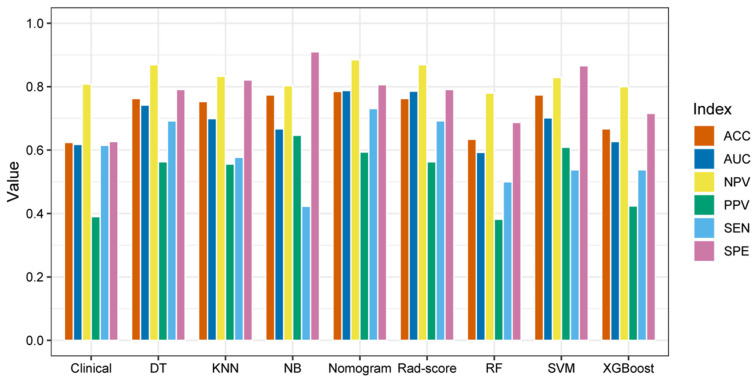
Bar plot of the performances of the nine prediction models in the validation set.

The predictive performances of the nine models, including seven machine learning classifiers, a clinical model, and a nomogram model, in the validation set are shown in Figure 10. The nomogram model has the highest AUC value (0.788), sensitivity (73.1%), and accuracy (78.5%), and NB has the highest specificity (91.0%). To sum up, the overall discrimination performance of the nomogram model was better than that of other models.

### 3.7. Clinical Application of the Prediction Models

**Figure 11:**
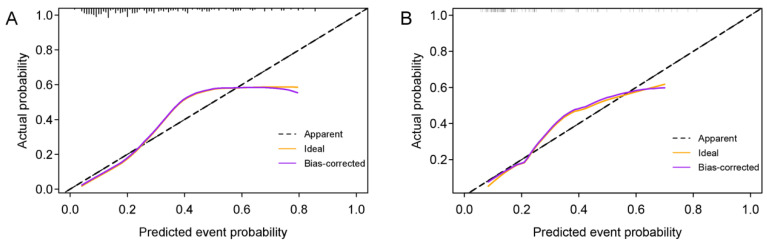
Calibration curves of the nomogram model in the training (**A**) and validation sets (**B**).

The calibration curve for the nomogram was tested using the Hosmer-Lemeshow test and yielded nonsignificant results due to both *p* values > 0.05 in the training and validation sets, showing good agreements between the observed and predicted results (Figure 11).

**Figure 12:**
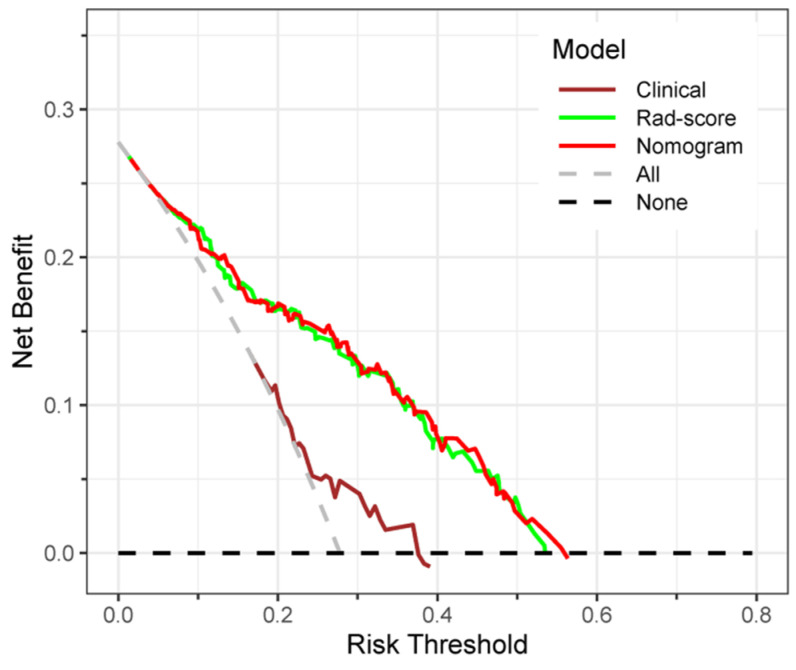
Decision curves of the models. If the risk threshold is less than 56.9%, the nomogram model will obtain more benefit than all treatment (assuming all breast cancer patients were HER2+) or no treatment (assuming all breast cancer patients were HER2−).

Decision curve analysis of the clinical, Rad-score and nomogram models is shown in Figure 12. The gray line represents the assumption that all lesions were HER2+ type. The black line represents the assumption that all lesions were HER2− type. If the threshold probability was less than 56.9%, using the nomogram would add more benefit (red line).

## 4. Discussion

Mineable data can be extracted from digital medical images by radiomics and analyzed to improve detection, diagnosis, staging, and prognosis prediction [20,21,22,24]. Ultrasound radiomics might be helpful to answer questions like what the molecular subtype of BC is, and this might affect the treatment strategy in patients with BC.

In our study, seven machine learning classifiers, such as KNN, LR, SVM, DT, NB, RF, and XGBoost, were established based on the Rad-score in the training set and tested in the time-independent validation set. Among them, the LR classifier with the AUC value of 0.786 performed the best, which might be that complex classifiers needed more training samples. Then the LR classifier was selected as the Rad-score model. The results indicated that the ultrasound-related Rad-score could predict the HER2+ status of patients with breast carcinoma. In addition, by establishing a nomogram model combining the Rad-score with clinical risk factors, we found that the nomogram model had significantly improved predictive performance compared with the model only involving clinical risk factors (AUC, 0.788 vs. 0.618, in the validation set) and slightly improved the ability compared with the Rad-score model (AUC, 0.788 vs. 0.786, in the validation set). The consistency between the nomogram model’s predicted probability of HER2 status and the actual results were evaluated by the calibration curve, and *p*-values in the training and validation sets were all > 0.05, which suggested that the stability of the model is fine. In addition, patients with BC could obtain a pronounced net benefit from the nomogram model when the threshold probability is less than 56.9%, which is shown in the decision curve analysis, demonstrating the good clinical utility of this model. The nomogram model could be potentially utilized as a routine tool to assist clinicians in preoperatively predicting HER2 status non-invasively.

In recent years, radiomics studies have mainly been carried out based on computer tomography or magnetic resonance imaging [19,20,21,22], demonstrating that radiomics features could reflect the heterogeneity of tumors and have become a reliable potential biomarker for improving diagnosis and treatment decisions. In recent radiomics studies on breast ultrasound imaging, researchers have mainly focused on the differential diagnosis of benign and malignant breast tumors [27,30,31], prediction of preoperative axillary lymph node metastasis [26,32,33], and prediction of molecular subtypes [28], with mixed findings that might be due to the heterogeneity of ultrasound machines, algorithms, and extracted features. The results of our study facilitate a possible clinical role for the nomogram model in the identification of HER2 status in BC, in accordance with the mentioned studies above carried out by ultrasound radiomics.

In the present study, the ultrasound images of breast carcinomas were analyzed by radiomics, and finally 12 features were screened out to calculate the radiomics score. A majority of the selected ultrasound radiomics features were wavelet-based features that were supposed to redisplay tumor characteristics hidden behind the speckle and show discriminative ability [32,34]. Among the 12 features, original_glszm_SmallAreaEmphasis revealed the strongest correlation with HER2+, while wavelet-LHL_glcm_Idn and wavelet-HLL_gldm_DependenceNonUniformityNormalized also showed a strong correlation. The relationship between the combinations of gray levels in the image parameters is calculated by glcm texture features, which have been widely used in many texture analysis applications and can reflect the internal spatial heterogeneity of the tumor lesions [35,36]. In the present study, glcm features extracted from an ultrasound image of BC were correlated with HER2 status. Radiomics features extracted from ultrasound image of BC could detect the invisible heterogeneity of tumors and were available to predict HER2 status in patients with BC.

Generally, one feature selection method is adopted in conventional radiomics analysis. In the study by Xu et al. [37], six features based on ultrasound radiomics were selected by the recursive feature elimination, and a random forest model including 90 trees was built for prediction of HER2 status, with the AUC of 0.780 and 0.740 in the training and validation sets. In order to reduce overfitting effectively, we used the ICC and Mann–Whitney U test for feature selection in the first step and LASSO regression in the second step, and we achieved better predictive performance with the LR classifier than the study by Xu et al., with AUC values of 0.804 and 0.786 in the training and validation sets, respectively. In addition, the statistical power of our study might be more robust because the sample size in our study was significantly larger than theirs (309 vs. 114).

A prior study by Wu et al. based on ultrasound radiomics developed models to predict the expression of molecular biomarkers of the mass type of breast ductal carcinoma in situ (DCIS) [29]. Based on 41 ultrasound radiomics features, they generated a model predictive of HER2+ type in BC patients with AUC values of 0.940 in the training set and 0.740 in the validation set. As the significantly reduced AUC value in the validation set and 41 ultrasound radiomics features (much more than 10% of the sample size of the training set) were selected to establish the model, we speculated that the overfitting problem should be taken into account. Moreover, in their study, only patients with a mass type of DCIS were enrolled, whereas in this study, tumors such as invasive ductal carcinoma, invasive lobular carcinoma, and mucinous breast carcinoma were included, which expanded the range of tumor types. Furthermore, the sample size of their retrospective study was much smaller than ours (116 vs. 309). Hence, compared with the study by Wu et al., a major highlight in our study was the larger sample size and diversity of tumor types, which might increase the universality of the nomogram model. We obtained a higher AUC value compared to the aforementioned studies with regards to prediction of HER2 status by using radiomics and a machine-learning algorithm [29,37]. The most probable explanation for this is that we adopted seven machine learning classifiers to develop seven prediction models and selected the one with the highest AUC value. Furthermore, the nomogram model combining the Rad-score with the clinical risk factor of tumor size was constructed and achieved better predictive performance than the LR classifier.

Despite the significance of the present research, there are several shortcomings in our study. Firstly, the prediction model based on ultrasound radiomics features was established and tested for identifying between HER2+ and HER2− BC in a single hospital with only 216 patients in the training set and 93 patients in the validation set. In addition, as all data was collected retrospectively and limited to Chinese patients, bias was inevitable. Therefore, further prospective studies need to involve a larger patient population and perform multicenter external validation. Secondly, in our study, the extraction of radiomics features required time-consuming tumor boundary segmentation and human-defined features, and we believe that a deep learning algorithm might accurately and automatically detect, segment, and achieve more objective results [38,39]. Thirdly, only gray-scale ultrasound images were adopted to develop the radiomics model, and other types of images like elastosonography or color Doppler ultrasound might be taken into account for multi-modal imaging to improve the predictive performance. Finally, radiomics studies based on gray-scale ultrasound images still lack reproducibility, as researchers always select different ultrasound images of the same lesion for radiomics analysis. Three-dimensional ultrasound images for feature extraction might be more objective than the conventional two-dimensional images, which could be considered in future studies.

## 5. Conclusions

In summary, the Rad-score model performs best among the seven classifiers. The nomogram model based on Rad-score and tumor size has slightly better predictive performance than the Rad-score model, and it has the potential to be utilized as a routine modality for preoperatively determining HER2 status in BC patients non-invasively. However, further studies with a prospective design and a larger population are required to validate the conclusions.

## Figures and Tables

**Table 1:** The baseline characteristics of the enrolled patients in the training and validation sets.

Characteristic	Total Set (*n* = 309)	Training Set (*n* = 216)	Validation Set (*n* = 93)	*p*-Value
Age (year, mean ± SD)	52.88 ± 10.96	53.61 ± 10.98	51.18 ± 10.76	0.073
Size (mm, mean ± SD)	24.58 ± 11.06	25.25 ± 11.03	23.02 ± 11.03	0.106
Tumor location				0.480
Right lobe	165	112	53	
Left lobe	144	104	40	
BI-RADS				0.297
4A	46	29	17	
4B	116	79	37	
4C	81	63	18	
5	66	45	21	
ER				0.973
Positive	228	160	68	
Negative	91	56	25	
PR				0.597
Positive	188	134	54	
Negative	121	82	39	
HER2				1.000
Positive	86	60	26	
Negative	223	156	67	
Histologic type				0.581
Invasive ductal	259	184	75	
Invasive lobular	14	9	5	
Other	36	23	13	
Ultrasound equipment				0.636
Siemens Acuson S2000	246	174	72	
LOGIQ E9	63	42	21	
US-reported LN				0.875
Metastasis positive	130	92	38	
Metastasis negative	179	124	55	
Pathology-reported LN				0.868
Metastasis positive	170	120	50	
Metastasis negative	139	96	43	
Ki-67 (%, mean ± SD)	28.52 ± 22.16	28.16 ± 21.96	29.38 ± 22.72	0.663
Radiomics score (median, IQR)	−0.0097 (−0.0975, 0.0794)	−0.0099 (−0.1030, 0.0787)	−0.0029 (−0.0883, 0.0808)	0.678

ER, estrogen receptor; PR, progesterone receptor; HER2, human epidermal growth factor receptor 2; SD, standard deviation; IQR, interquartile range; LN, lymph node; US, ultrasound; BI-RADS, Breast Imaging Reporting and Data System.

**Table 2:** List of features with nonzero coefficients.

Image Type	Feature Class	Feature Name	Coefficient
original	shape	Elongation	−0.011322
original	glszm	SmallAreaEmphasis	−0.076092
wavelet-LHL	glcm	Idn	0.047259
wavelet-LHL	glszm	SmallAreaLowGrayLevelEmphasis	−0.013013
wavelet-LHH	glszm	HighGrayLevelZoneEmphasis	0.008385
wavelet-LHH	glszm	SizeZoneNonUniformityNormalized	0.005098
wavelet-HLL	firstorder	90Percentile	−0.020703
wavelet-HLL	glcm	JointEntropy	0.020412
wavelet-HLL	glszm	GrayLevelNonUniformityNormalized	−0.010225
wavelet-HLL	gldm	DependenceNonUniformityNormalized	−0.033653
wavelet-HLH	firstorder	Mean	−0.00703
wavelet-HHH	firstorder	Median	0.010776

**Table 3:** Rad-score for the training and validation sets.

Rad-Score	HER2− (Median, IQR)	HER2+ (Median, IQR)	*p*-Value
Training set	−0.0546 (−0.1303, 0.0338)	0.0838 (0.0336, 0.1523)	<0.001
Validation set	−0.0518 (−0.0985, 0.0394)	0.0936 (0.0185, 0.1623)	<0.001

IQR, interquartile range.

**Table 4:** Diagnostic performance of seven machine learning classifiers in training and validation sets.

	Training Set				Time-Independent Validation Set			
Model	AUC (95%CI)	SEN	SPE	ACC	AUC (95%CI)	SEN	SPE	ACC
LR	0.804 (0.742–0.865)	80.0%	70.5%	73.1%	0.786 (0.683–0.890)	69.2%	79.1%	76.3%
SVM	0.691 (0.622–0.760)	51.7%	86.5%	76.9%	0.702 (0.596–0.808)	53.8%	86.6%	77.4%
KNN	0.708 (0.641–0.776)	50.0%	91.7%	80.1%	0.699 (0.592–0.806)	57.7%	82.1%	75.3%
RF	1.000 (1.000–1.000)	100.0%	100.0%	100.0%	0.593 (0.480–0.706)	50.0%	68.7%	63.4%
DT	0.747 (0.680–0.814)	66.7%	82.7%	78.2%	0.742 (0.639–0.845)	69.2%	79.1%	76.3%
XGB	0.917 (0.872–0.963)	86.7%	96.8%	94.0%	0.627 (0.516–0.739)	53.8%	71.6%	66.7%
NB	0.655 (0.589–0.722)	40.0%	91.0%	76.9%	0.667 (0.564–0.770)	42.3%	91.0%	77.4%

DT, decision tree; RF, random forest; SVM, support vector machine; LR, logistic regression; NB, naive Bayes; KNN, K nearest neighbors; XGB, XGBboost; AUC, area under the curve; SEN, sensitivity; SPE, specificity; ACC, accuracy.

**Table 5:** P values for AUC comparison between any pair of models tested by the DeLong method in the validation set.

Model (AUC Value)	LR (0.786)	SVM (0.702)	KNN (0.699)	RF (0.593)	DT (0.742)	XGB (0.627)	NB (0.667)
LR (0.786)	1	-	-	-	-	-	-
SVM (0.702)	0.023	1	-	-	-	-	-
KNN (0.699)	0.054	0.955	1	-	-	-	-
RF (0.593)	0.004	0.164	0.101	1	-	-	-
DT (0.742)	0.124	0.317	0.225	0.021	1	-	-
XGB (0.627)	0.042	0.344	0.367	0.674	0.142	1	-
NB (0.667)	0.006	0.305	0.574	0.329	0.124	0.612	1

LR, logistic regression; KNN, K nearest neighbors; DT, decision tree; RF, random forest; SVM, support vector machine; NB, naive Bayes; XGB, XGBboost; AUC, area under the curve. The bold numbers (<0.05) mean statistical difference.

**Table 6:** Comparison of the clinical features between the HER2+ and HER2− BC groups in the training set.

	Training Set (*n* = 216)		
Clinical Feature	HER2− (*n* = 156)	HER2+ (*n* = 60)	*p*-Value
Age (year, mean ± SD)	54.04 ± 11.78	52.47 ± 8.55	0.279
Tumor location			0.673
Right	79	33	
Left	77	27	
Tumor size (mm, mean ± SD)	24.21 ± 10.90	27.93 ± 11.02	0.028
US equipment			0.064
Siemens Acuson S2000	131	43	
LOGIQ E9	25	17	
US-reported LN			0.550
Metastasis positive	64	28	
Metastasis negative	92	31	
Rad-score (median, IQR)	−0.0546 (−0.1303, 0.0338)	0.0838 (0.0336, 0.1523)	*p* < 0.001

SD, standard deviation; LN, lymph node; US, ultrasound; IQR, interquartile range.

**Table 7:** Predictive performances of the models identifying HER2+ status in patients with BC.

	Training Set				Time-Independent Validation Set			
Model	AUC (95%CI)	SEN	SPE	ACC	AUC (95%CI)	SEN	SPE	ACC
Clinical	0.594 (0.509–0.679)	48.3%	69.9%	63.9%	0.618 (0.485–0.751)	61.5%	62.7%	62.4%
Rad-score	0.804 (0.742–0.865)	80.0%	70.5%	73.1%	0.786 (0.683–0.890)	69.2%	79.1%	76.3%
Nomogram	0.804 (0.742–0.866)	81.7%	71.8%	74.5%	0.788 (0.685–0.891)	73.1%	80.6%	78.5%

AUC, area under the curve; SEN, sensitivity; SPE, specificity; ACC, accuracy.

## Data Availability

The data presented in this study are available on request from the corresponding author.

## Supplementary Materials

The following supporting information can be downloaded at: https://www.mdpi.com/article/10.3390/diagnostics12123130/s1. Data S1: The ultrasound radiomics extraction settings; Data S2: The morphological characteristics of the randomly selected 50 lesions for ICC assessment; Data S3: The corresponding fitting formula for calculating the Rad-score.
