# Supplementary material for: Development and Validation of an Ultrasound-Based Radiomics Nomogram for Identifying HER2 Status in Patients with Breast Carcinoma
Source: Diagnostics (Basel). 2022 Dec 12;12(12):3130. doi: 10.3390/diagnostics12123130 (PMC9776855; doi:10.3390/diagnostics12123130)
Supplement: Supplementary file 1 [file diagnostics-12-03130-s001.zip › Data S1 The ultrasound radiomics extraction settings.pdf]

#Details related to feature extraction

```
# This is an example of a parameters file
# It is written according to the YAML-convention (www.yaml.org) and is checked by the code for
consistency.
# Three types of parameters are possible and reflected in the structure of the document:
#
# Parameter category:
#     Setting Name: <value>
#
# The three parameter categories are:
# - setting: Setting to use for preprocessing and class specific settings. if no <value> is specified,
the value for
#     this setting is set to None.
# - featureClass: Feature class to enable, <value> is list of strings representing enabled features. If
no <value> is
#     specified or <value> is an empty list ('[]'), all features for this class are enabled.
# - imageType: image types to calculate features on. <value> is custom kwarg settings (dictionary).
if <value> is an
#     empty dictionary ('{}'), no custom settings are added for this input image.
#
# Some parameters have a limited list of possible values. Where this is the case, possible values
are listed in the
# package documentation

# Settings to use, possible settings are listed in the documentation (section "Customizing the
extraction").
setting:
  binWidth: 25
  label: 1
  interpolator: 'sitkBSpline' # This is an enumerated value, here None is not allowed
  resampledPixelSpacing: # This disables resampling, as it is interpreted as None, to enable it,
specify spacing in x, y, z as [x, y , z]
  weightingNorm: # If no value is specified, it is interpreted as None

# Image types to use: "Original" for unfiltered image, for possible filters, see documentation.
imageType:
  Original: {} # for dictionaries / mappings, None values are not allowed, '{}' is interpreted as an
empty dictionary
  LoG:
    # Because of resampling to (3, 3, 3), the use of sigmas < 3 mm is not recommended.
    sigma: [3.0, 5.0]
  Wavelet: {}
# Featureclasses, from which features must be calculated. If a featureclass is not mentioned, no
features are calculated
```

for that class. Otherwise, the specified features are calculated, or, if none are specified, all are calculated (excluding redundant/deprecated features).

featureClass:

redundant Compactness 1, Compactness 2 an Spherical Disproportion features are disabled by default, they can be

enabled by specifying individual feature names (as is done for glcm) and including them in the list.

shape:

firstorder: [] # specifying an empty list has the same effect as specifying nothing.

glcm: # Disable SumAverage by specifying all other GLCM features available

- 'Autocorrelation'
- 'JointAverage'
- 'ClusterProminence'
- 'ClusterShade'
- 'ClusterTendency'
- 'Contrast'
- 'Correlation'
- 'DifferenceAverage'
- 'DifferenceEntropy'
- 'DifferenceVariance'
- 'JointEnergy'
- 'JointEntropy'
- 'Imc1'
- 'Imc2'
- 'Idm'
- 'Idmn'
- 'Id'
- 'Idn'
- 'InverseVariance'
- 'MaximumProbability'
- 'SumEntropy'
- 'SumSquares'

glrlm: # for lists none values are allowed, in this case, all features are enabled

glszm:

gldm: # contains deprecated features, but as no individual features are specified, the deprecated features are not enabled
