# Supplementary material for: Development and Validation of an Ultrasound-Based Radiomics Nomogram for Identifying HER2 Status in Patients with Breast Carcinoma
Source: Diagnostics (Basel). 2022 Dec 12;12(12):3130. doi: 10.3390/diagnostics12123130 (PMC9776855; doi:10.3390/diagnostics12123130)
Supplement: Supplementary file 1 [file diagnostics-12-03130-s001.zip › Data S2 The morphological characteristics of the randomly selected 50 lesions for ICC assessment.pdf]

Supplementary table The morphological characteristics of the 50 randomly selected lesions

**Morphological feature**	**Number**
Internal echo	
Homogeneous	2
Not homogeneous	48
Mass margin	
Circumscribed	3
Not circumscribed	47
Mass shape	
Round or oval	2
Irregular	48
Composition	
Cystic-solid mixture	1
Solid, dominantly solid	49
Echogenicity	
Isoechoic	1
Hypoechoic	49
