# Supplementary material for: Development and Validation of an Ultrasound-Based Radiomics Nomogram for Identifying HER2 Status in Patients with Breast Carcinoma
Source: Diagnostics (Basel). 2022 Dec 12;12(12):3130. doi: 10.3390/diagnostics12123130 (PMC9776855; doi:10.3390/diagnostics12123130)
Supplement: Supplementary file 1 [file diagnostics-12-03130-s001.zip › Data S3 The corresponding fitting formula for calculating the Rad-score.pdf]

Rad-score=
-0.076092*original_glszm_SmallAreaEmphasis +
-0.033653*wavelet-HLL_gldm_DependenceNonUniformityNormalized+
-0.020703*wavelet-HLL_firstorder_90Percentile+
-0.013013*wavelet-LHL_glszm_SmallAreaLowGrayLevelEmphasis+
-0.011322*original_shape_Elongation+
-0.010225*wavelet-HLL_glszm_GrayLevelNonUniformityNormalized +
-0.00703*wavelet-HLH_firstorder_Mean+
0.005098*wavelet-LHH_glszm_SizeZoneNonUniformityNormalized+
0.008385*wavelet-LHH_glszm_HighGrayLevelZoneEmphasis+
0.010776*wavelet-HHH_firstorder_Median+
0.020412*wavelet-HLL_glcm_JointEntropy+
0.047259*wavelet-LHL_glcm_Idn
